# Big Data and Network Biology

**DOI:** 10.1155/2014/836708

**Published:** 2014-06-15

**Authors:** Shigehiko Kanaya, Md. Altaf-Ul-Amin, Samuel Kuria Kiboi, Farit Mochamad Afendi

**Affiliations:** ^1^Nara Institute of Science and Technology (NAIST), Nara 630-0192, Japan; ^2^University of Nairobi, Nairobi 00100, Kenya; ^3^Bogor Agricultural University, Java Barat 16680, Indonesia

With the data deluge caused by the recent high throughput experiments in molecular biology emerged the popular topics such as big data biology and network biology aiming at understanding life as a system by integrating and applying knowledge and facilities of different branches of science including mathematics, physics, statistics, chemistry, computer science, and information technology. Naturally, the spectrum of topics under big data and network biology is widespread and the present special issue is not an exhaustive representation of the subject. Nonetheless the articles selected for this special issue represent recent trends and versatile knowledge concerning the title topic, that we have the pleasure of sharing with the readers. Data-intensive sciences like contemporary biology consist of three basic activities: capture, curation, and analysis. Being in the bioinformatics domain, this special issue mainly focuses on analysis; that is, it contains articles about novel tools and methodologies for data analysis and mining and review articles describing databases, tools, and algorithms useful for curation and analysis of biological data.

This special issue contains fifteen papers. Three papers discuss software tools for analyzing different “omics” data. Three other papers are review papers discussing versatile aspects of systems biology. Two of the papers present biomedical text mining approaches. The other seven papers are methodology articles related to genomics, transcriptomics, proteomics, metabolomics, and herbal medicines.

The paper “*Systems biology in the context of big data and networks,*” which is a review paper, gives an overview of the progress in big data biology and data handling and also introduces some applications of networks and multivariate analysis in systems biology.

The paper “*AmalgamScope: merging annotations data across the human genome*” presents a new interactive software tool developed to assist scientists with annotation of the human genome and in particular the integration of the annotations from multiple data types, using gene identifiers and genomic coordinates. Supported platforms include next-generation sequencing and microarray technologies.

The paper “*Integration of residue attributes for sequence diversity characterization of terpenoid enzymes*” first determined important metrics describing the biochemical and physical attributes of amino acids. It utilized random forest algorithm to reduce redundancies in the amino acid index. This research contributes a different mechanism toward handling protein sequences. It especially quantifies the sequence information to numerical scale and thus facilitates the application of computational algorithms.

The paper entitled “*OWL reasoning framework over big biological knowledge network*” presents a general OWL (web ontology language) reasoning framework to systematically study and reveal the implicit relationships among biological entities from big biological networks. In their experiment, the authors focused on association between traditional Chinese medicine (TCM) and Western medicine (WM). The derived associations are useful for biologists to promote the development of novel drugs and also for modernization of TCM.

The paper entitled “*A knowledge-driven approach to extract disease-related biomarkers from the literature*” developed a text mining approach to extract a dataset of biomarkers related to diseases covering all therapeutic areas by exploiting a large literature database. Additionally, this work presents a bibliometric analysis of the journals reporting biomarker related information during the last 40 years.

The paper “*Integrated analysis of gene network in childhood leukemia from microarray and pathway databases*” delineates differential responses of acute lymphoblastic leukemia (ALL) subtypes, B-ALL and T-ALL, to glucocorticoids (GCs) treatment by identifying the differences among biological processes, molecular pathways, and interaction networks that emerge from the action of GCs.

The paper “*Tools and databases of the KOMICS web portal for preprocessing, mining, and dissemination of metabolomics data*” presents a metabolomics web portal which includes the tools and databases for preprocessing, mining, visualization, and publication of metabolomics data. Metabolomics research is increasingly utilized for applications such as disease diagnostics, biomarker discovery, and assessment of food quality.

The paper entitled “*Supervised clustering based on DPClusO: prediction of plant-disease relations using Jamu formulas of KNApSAcK database*” proposes a new approach to predict the relation between effective therapeutic plant and disease using network analysis and supervised clustering based on the ingredient data of Indonesian herbal medicines called Jamu. Scientific analysis of traditional medicines is important because such medicines have been developed through hundreds of years of human experience.

The paper entitled “*A novel feature selection strategy for enhanced biomedical event extraction using the Turku system*” proposed a method to enhance the performance of Turku Event Extraction System (TEES). This work developed and applied an accumulated effect evaluation (AEE) algorithm to identify important features for text-mining classifiers.

The paper entitled “*A novel bioinformatics method for efficient knowledge discovery by BLSOM from big genomic sequence data*” constructed oligonucleotide BLSOM corresponding to a wide range of vertebrate genomes and detected differences between human and mouse genomes. Due to its high classification and visualization power, BLSOM recognized the species-specific key combination of oligonucleotide frequencies in each genome, described as “genome signature.”

The paper titled “*Applied graph-mining algorithms to study biomolecular interaction networks*” is a review paper. Graph comparison and module detection are the two most commonly used strategies for analyzing PPI or other biological networks. This paper summarizes the current literature on graph kernel and graph alignment methods for graph comparison, as well as a variety of module detection approaches including seed-and-extend, hierarchical clustering, optimization-based, probabilistic, and frequent subgraph methods.

The paper “*An unsupervised approach to predict functional relations between genes based on expression data*” first digitizes the log-ratio type gene expression data to a matrix consisting of 1, 0, and −1 indicating highly expressed, no major change, and highly suppressed conditions, respectively, for genes. For each pair of genes, a probability density mass function table is constructed indicating nine joint probabilities and those probability values were intelligently utilized to find functional relations between genes.

The paper entitled “*Survey of network-based approaches to research of cardiovascular diseases*” is a review article on cardiovascular diseases (CVDs) which are the leading health problems worldwide. Biomolecular interaction networks generated from available data are excellent platforms for understanding linkage of all processes within a living cell, including processes that underlie diseases. This work reviewed approaches that explore and use relationships between topological properties of biological networks and mechanisms underlying CVDs.

The paper “*Essential functional modules for pathogenic and defensive mechanisms in Candida albicans infections*” adopted a systems biology approach to construct the early-stage and late-stage protein-protein interaction (PPI) networks for both* C. albicans *and zebrafish and, by comparing those PPI networks, identified several critical functional modules in both pathogenic and defensive mechanisms.

The paper entitled “*Visualization of genome signatures of eukaryote genomes by batch-learning self-organizing map with a special emphasis on Drosophila genomes*” generated the BLSOMs for tetra- or pentanucleotide composition in approximately one million sequence fragments derived from 101 eukaryote genomes. BLSOM recognized phylotype-specific characteristics (e.g., key combinations of oligonucleotide frequencies) in the genomic sequences, by clustering the sequences without adding any prior information regarding the species.

